# Açaí (*Euterpe oleracea* Mart) Consumption and Prevention of Chronic Diseases: Is There an Association? A Preliminary Study

**DOI:** 10.1155/2020/5782485

**Published:** 2020-06-01

**Authors:** Dulcelena Ferreira Silva, Marcos Antonio Custódio Neto da Silva, Gabrielle Meirelles Rodrigues, Flávia Castello Branco Vidal, Maria do Carmo Lacerda Barbosa, Luciane Maria Oliveira Brito, Geusa Felipa de Barros Bezerra, Walbert Edson Muniz Filho, Kátia Regina Assunça˜o Borges, Ivone Garros Rosa, Joa˜o Ernesto de Carvalho, Maria do Desterro Soares Branda˜o Nascimento

**Affiliations:** ^1^Morphology Department, Federal University of Maranha˜o, Sa˜o Luís, Maranha˜o, Brazil; ^2^Post-Graduate Program in Internal Medicine, Medical Science School, State University of Campinas, Campinas, Sa˜o Paulo, Brazil; ^3^Federal University of Maranha˜o, Sa˜o Luís, Maranha˜o, Brazil; ^4^Morphology Department, Post-Graduate Program in Adult Health, Federal University of Maranha˜o, Sa˜o Luís, Maranha˜o, Brazil; ^5^Medicine I Department, Federal University of Maranha˜o, Sa˜o Luís, Maranha˜o, Brazil; ^6^Medicine III Department, Post-Graduate Program in Adult Health, Federal University of Maranha˜o, Sa˜o Luís, Maranha˜o, Brazil; ^7^Pathology Department, Post-Graduate Program in Adult Health, Federal University of Maranha˜o, Sa˜o Luís, Maranha˜o, Brazil; ^8^Pathology Department, Federal University of Maranha˜o, Sa˜o Luís, Maranha˜o, Brazil; ^9^Pathology Department, Health and Environment Post-Graduate Program, Federal University of Maranha˜o, Sa˜o Luís, Maranha˜o, Brazil; ^10^Pharmaceutic Science School, Post-Graduate Program in Internal Medicine, State University of Campinas, Campinas, Sa˜o Paulo, Brazil

## Abstract

**Background:**

Flavonoids from a variety of fruits, including açaí, have beneficial antioxidant activity in several diseases, including cancer. Breast cancer is the second most prevalent cancer among Brazilian women. Studies have shown the action of flavonoids on neoplastic cells, as well as on diabetes and neurodegenerative and cardiovascular diseases.

**Objective:**

To analyze the relationship between the consumption of açaí and the presence of chronic diseases in women residing in the rural area of São Luís, Maranhão.

**Methods:**

A convenience sample of 150 women residing in the Maracanã neighborhood in São Luís, Maranhão, was used; the collected data included sociodemographic characteristics, habits, sexual and reproductive history, consumption of açaí, and history of cancer and other chronic diseases. The sample was divided into women who consumed açaí at least once a week (cases) and women who did not consume açaí (controls). Statistical analysis was performed to assess the relationships between those variables and the consumption of açaí.

**Results:**

A total of 141 women (94%) consumed açaí. Among these, 79.3% were aged between 20 and 50 years, 78.67% were farmers or housewives, 64.67% were Pardo (mixed race), 76.67% were nonsmokers, 70% were not receiving hormonal therapy, 40.67% had already undergone mammography, 28% had already undergone breast ultrasound, and 27.33% had a family history of cancer, with breast cancer being the second most prevalent cancer. There was a higher prevalence of hypertension among women who did not consume açaí than that among those who did; however, previous cancer, family history of cancer, heart disease, and diabetes were more prevalent among the consumers of açaí. There were no statistically significant relationships.

**Conclusion:**

Flavonoids are known to have a beneficial effect on some types of neoplastic cells and other diseases; therefore, larger studies are necessary to better evaluate the beneficial effects of consuming foods containing flavonoids on these diseases.

## 1. Introduction

Açaí (*Euterpe oleracea* Mart.) is a typical and popular fruit of the Amazon region that has gained importance in recent years because of its health benefits, which are associated with its phytochemical composition and antioxidant capacity [1]. It is primarily found in states of the Northern region of Brazil. The state of Pará is the largest producer of açaí, followed by the states of Maranhão, Amapá, Acre, and Rondônia [2].

Samples of açaí have been studied and shown to contain cyanidin-3-glucoside, cyanidin-3 sambubioside, cyanidin-3-rutinoside, and peonidin 3-rutinoside. Açaí has a high concentration of anthocyanins (1.02 g/100 g of dry solids), whose content in fruits is related to the prevention and reduction in the risk of cancer and cardiovascular and circulatory diseases through the scavenging of free radicals [3].

Experimental studies with transgenic mice and models of cancer mediated by carcinogens such as radiation and agents derived from exogenous or endogenous sources have shown that some phytochemicals consumed in the diet have a protective effect against cancer. This protection provided by dietary phytochemicals is the result of the induction of cellular defense mechanisms, including antioxidants, detoxification, activation of anti-inflammatory enzymes, and signaling pathways that lead to cell cycle arrest and/or cell death [4].

Cancer is a growing health problem worldwide, resulting from the increase in life expectancy and urbanization and subsequent changes to the environment [5]. Breast cancer is the most common type of cancer among women in Brazil and worldwide, after nonmelanoma skin cancer, with approximately 57,960 new cases (28.1%) and an incidence of 56.2 cases per 1,000 people in 2016 [6].

Cancer-preventing phytochemicals, especially flavonoids, have been shown to suppress or prevent the progression of cancer by a variety of mechanisms. The intake of some types of flavonoids may contribute to the prevention of breast cancer [7], and a potent chemopreventive agent from the açaí extract has been shown in experimental studies to be useful in estrogen-dependent breast cancer treatment (*Euterpe oleracea* Mart.) [5].

Plants contain natural compounds with important bioactive properties, which might be alternatives to the currently used medications and lead to the discovery of new drugs [8, 9]. The consumption of açaí is associated with the prevention and reduction in the risk of developing cancer and cardiovascular and circulatory diseases, in addition to the promotion of healthy aging and reduction of oxidative damage [10].

Thus, the objective of the present study was to analyze the relationship between the consumption of the açaí fruit and the presence of chronic diseases in women residing in the rural area of São Luís, Maranhão.

## 2. Methods

### 2.1. Study Type and Area

This cross-sectional, retrospective, and analytical study assessed the prevalence of açaí intake among women in São Luís, Maranhão, and its association with chronic diseases.

The study was conducted in the municipality of São Luís among women residing in the Maracanã Ecological Park, an Environmentally Protected Area (EPA), in which the Juçara Park is located [11].

### 2.2. Study Population

The population selected for this study was a convenience sample with a calculated sample size of 150 individuals aged between 12 and 80 years. The study period was between November 2014 and February 2015.

The inclusion criteria were women who resided in the Maracanã Ecological Park who provided their informed consent for their inclusion in the study.

The exclusion criteria included women residing in the Maracanã Ecological Park with mental impairments that could hinder their understanding of the questions on the specific questionnaire and women who refused to provide their informed consent.

### 2.3. Sample

The sample was composed of 141 women residing in the Maracanã Ecological Park who consumed açaí at least once a week (cases) and nine women who did not consume açaí (controls).

### 2.4. Data Collection

Data collection was performed between November 2014 and February 2015.

Interviews were conducted using a structured epidemiological questionnaire that included information on sociodemographic characteristics, habits, sexual and reproductive history, consumption of açaí, and history of cancer.

### 2.5. Statistical Analysis

The data were entered into an electronic database and analyzed using Stata/SE 9.0 for Windows (Stata Corporation, College Station, Texas, USA). The categorical variables were expressed as absolute and percentage values and the numerical variables as means and standard deviations.

Chi-square test or Fisher's exact test was used to assess the relationship between categorical variables, and two-sided *t*-tests were used for comparison of numerical variables (age).

In all tests, the significance level (*α*) was set at 5%; i.e., the results were considered significant for *p* < 0.05.

### 2.6. Ethical Considerations

This study was submitted to and approved by the Research Ethics Committee of the University Hospital of the Federal University of Maranhão (number 814.669/2014). The women included in the study provided their informed consent (resolution 466/12 of the National Health Council and its complementary resolutions).

## 3. Results

The study included 150 women, of which 0.67% (1/150) were less than 20, 25.33% (38/150) were between 20 and 30, 33.33% (50/150) were between 31 and 40, 15020.67% (31/150) were between 41 and 50, 10.67% (16/150) were between 51 and 60, 6.00% (9/150) were between 61 and 70, and 3.33% (5/150) were between 71 and 80 years of age. The patients' ages ranged between 12 and 80 years (mean age of 40.49 years, SD ± 13.23). There was no statistically significant difference in age between the group that consumed açaí (141 women) and the control group (9 women). With regard to years of schooling, 6.67% (10/150) were illiterate women and 9.34% (14/150), 27.33% (41/150), 43.33% (65/150), and 12.67% (19/150) of women had studied for 1 to 3, 4 to 7, 8 to 11, and more than 11 years, respectively. With regard to occupation, 40.67% (70/150) were housewives, 32% (48/150) were farmers, 14.67% (22/150) were merchants, 4.66% (7/150) were retired, and 2.00% (3/150) were students. The distributions according to marital status were as follows: 36.67% (55/150) were single; 32.67% (49/150) were married; 22.00% (33/150) lived in cohabitation, 6.66% (10/150) were widows, and 2.00% (3/150) were separated. With regard to ethnicity, 64.67% (97/150) were Pardo Brazilian, 20.67% (31/150) were Afro-Brazilian, 13.33% (20/150) were White Brazilian, 0.67% (1/150) were indigenous, and 0.66% (1/150) were Asian Brazilian. The analysis of family income showed that 15.34% (23/150) received half of the minimum salary per month, 48.00% (72/150) received one minimum salary per month, 21.33% (32/150) received two minimum salaries per month, 0–67% (1/150) received three minimum salaries per month, 11.33% (17/150) received more than three minimum salaries per month, and 3.33% (5/150) did not answer (Table 1).

Most women were nonsmokers (76.67%; 115/150) and 6.00% (9/150) were former smokers; however, most of the former and current smokers (35 women) had been or were smokers for 10 years or more (57.15%; 20/35). In the sample, 54.67% (82/150) of women were not alcoholics, 42.00% (63/150) consumed alcoholic beverages, and 3.33% (5/150) were former alcoholics (Table 1).

With regard to açaí intake, 94.00% (141/150) consumed the fruit at least once a week and 6.00% (9/150) did not consume it. The majority of those who consumed açaí (77.30%; 109/141) had been consuming it for more than 10 years, 51.77% (73/141) consumed açaí at least three times a week, and 86.52% (122/141) consumed it in the form of juice (Table 2).

With regard to reproductive characteristics, the majority of women (70.00%; 105/150) had attained menarche between 11 and 14 years of age and had not yet entered menopause (76.00%; 114/150). Moreover, 89.33% (134/150) had been pregnant, with the majority (52.00%; 78/150) having had two to four full-term pregnancies, 91.33% (137/150) had not had preterm pregnancies, 83.33% (125/150) never had an abortion, and 82.67% (124/150) had breastfed (Table 3). Hormonal therapy was not used by 70.00% (105/150) of the women, and most women who reported using hormonal therapy (57.78%; 26/45) used oral contraceptives only. Most women (88.67%; 133/150) did not have a history of breast trauma.

In the sample, 40.67% (61/150) of the women had undergone mammography, with 50.82% of these (31/61) having undergone an exam approximately one year before, 39.34% (24/61) having undergone an exam two or more years before, and 9.84% (6/61) not knowing when they had undergone an exam. The diagnostic result was Category I for most of these women (59.01%; 36/61). Most women (72.00%; 108/150) had not undergone a breast ultrasound. Among those who had, most (54.76%; 23/42) had undergone an exam approximately one year before, 35.71% (15/42) had undergone an exam two or more years before, and 9.52% (4/42) did not answer. Most women (85.71%; 36/42) had a Category I result. The results of the diagnostic exams were subjected to statistical analysis for correlation with açaí intake, with no statistically significant correlations (Table 4).

The assessment of comorbidities showed that 13.33% (20/150) of the women were diabetic, 6.67% (10/150) had heart disease, and 22.67% (34/150) were hypertensive. Statistical analysis revealed no statistically significant relationships between açaí consumption and the analyzed parameters (Table 5).

History of cancer revealed that 1.33% (2/150) had had cancer previously and the remaining women (98.67%; 148/150) had not and that 7.33% (11/150) had breast nodules. With regard to a family history of cancer, 27.33% (41/150) had cancer in the family. Of these, 31.71% (13/41) had a history of cancer in first-degree relatives (mother, father, and siblings), whereas 68.29% (28/41) had a history of cancer in second-degree relatives (uncles, grandparents, and cousins). The types of cancer in the family history included uterine (31.7%; 13/41), breast (26.83%; 11/41), ovarian (9.75%; 4/41), stomach, liver, bowel, prostate and penis cancers (each accounting for 4.88%; 2/41), and bone and lung cancers (each accounting for 2.44%; 1/41). One participant (2.44%; 1/41) did not answer this item. There were no statistically significant relationships between a previous history of cancer, presence of breast nodules, and family history of breast cancer and other types of cancer and açaí consumption (Table 5).

## 4. Discussion

Diets rich in fruits and vegetables have been reported to increase the antioxidant capacity of plasma. In addition to containing vitamins C and E and beta-carotene, these foods contain phenolic compounds with antioxidant properties [12]. Dietary flavonoids are a large family of bioactive phenolic compounds that occur naturally in plant-based foods and are present in significant amounts in several fruits, vegetables, grains, herbs, and commonly consumed drinks [13].

*Euterpe oleracea* Mart. is a monocotyledonous species indigenous of the floodplain forests of the Amazon region, particularly in Venezuela, Colombia, Ecuador, The Guianas, Suriname, and Brazil (mainly in the states of Amazonas, Amapá, Pará, Maranha˜o, Rondônia, Acre, and Tocantins). It is known by the population as açaí, “açaí do pará,” “açaí do baixo amazonas,” “açaí de touceira,” “açaí de planta,” “juçara,” and “juçara de touceira” [14, 15]. The two latter designations are fairly common in the state of Maranhão.

The fruit has a rounded shape, a purplish-black color, is approximately 25 mm in diameter, and contains only one large seed. The analysis of the phenolic compounds in açaí showed the presence of anthocyanin 3-glucoside, ferulic acid, epicatechin, p-hydroxybenzoic acid, gallic acid, protocatechuic acid, catechin, ellagic acid, vanillic acid, p-coumaric acid, and gallotannin [12].

The intake of fruits and vegetables has been associated with a reduction in the risk of cancer in humans, particularly breast cancer. Dietary flavonoids, a group comprising more than 5,000 different polyphenolic compounds, have been identified as components of fruits and vegetables with the potential to prevent cancer [16].

The high antioxidant capacity of açaí has been confirmed in several *in vitro* studies. Moreover, açaí was shown to have a higher antioxidant capacity than that of other fruits with antioxidant potential, including cranberries, blueberries, and strawberries. The antioxidant activity of açaí supplementation has been examined in *in vivo* studies conducted with mice. After six weeks of açaí consumption, there was an increase in the antioxidant activity of paraoxonase 1 (PON-1) in rats that received normal or cholesterol-enriched diets [17].

Epidemiological studies and systematic analyses suggest that diets rich in fruits and vegetables are associated with a reduction in the risk of developing cancer, especially cancers of epithelial origin such as mouth, colon, rectum, lung, and breast cancers [16]. However, in the case of chronic diseases, because it takes a long time (usually decades) for symptoms to manifest and because of the lack of animal models, epidemiological studies have been the key tool for establishing associations between the intake of food components and its subsequent effects on health [18].

Therefore, the objective of the present study was to analyze the relationship between açaí consumption and chronic diseases. For this purpose, a survey was conducted to collect data to assess this relationship. The results showed a high prevalence of açaí consumption among the women in the sample (94.00%; 141/150), most of which (77.30% or 109/141) had consumed açaí for more than 10 years. Despite this habit, there were no statistically significant differences relative to the control group (6.00%; 9/150) with regard to comorbidities, previous history of cancer, family history of cancer, changes in imaging exams (mammography and breast ultrasound), and presence of breast nodules. The prevalence of comorbidities among the açaí consumers was higher than that among the nonconsumers (with the exception of the prevalence of hypertension, which was higher in the group of nonconsumers). Breast cancer was the second most prevalent type of cancer included in the family history of cancer. In fact, several experimental studies have evaluated the effects of flavonoids present in extracts of açaí and other fruits on breast cancer cells [5, 7, 16, 19].

Our study evidenced a tendency of açaí to reduce diabetes and hypertension in women.

Açaí is rich in polyphenols that have some antidiabetic effects [20]. They can induce satiety, modulate the digestive processes and carbohydrate absorption, and modulate hormonal processes related to sugar intake [21–23].

de Bem et al. evidenced that association of exercise and the açaí seed extract in rats improved diabetic complications. Açaí reduced blood glucose, insulin resistance, leptin and IL-6 levels, lipid profile, and vascular dysfunction [24].

Regarding antihypertensive effects of açaí, da Costa et al. evidenced that administration of the açaí seed extract (200 mg/kg) increased systolic blood pressure, decreased urea and creatinine levels, and prevented oxidative damage in animals [25].

Polyphenols from the seed of acai increase endothelial nitric oxide production leading to endothelium-dependent relaxation, reduction of reactive oxygen species, and regulation of key targets in hypertension [26].

This study is the first study to correlate the açaí intake and chronic disease prevention in Sa˜o Luís, MA, Brazil. The sample size was not too great, and a small control group could be difficult for statistical analysis; the results were not significant.

Thus, studies on the relationship between the intake of fruits and vegetables rich in flavonoids and the risk of developing chronic diseases, including cancer, are of great value for the international literature and for the advancement of cancer treatment and prevention, especially in the case of cancers that are more prevalent and associated with higher morbidity, such as breast cancer in Brazil and worldwide. Further studies need to be performed to evaluate the relationship between açaí intake and chronic diseases.

## Figures and Tables

**Table 1:** Sociodemographic characteristics of women residing in the Maracanã neighborhood, São Luís, MA.

Variables	Açaí consumption		*p*
	No *f* (%)	Yes *f* (%)	
*Years of schooling*			0.39
Illiterate	2 (22.22)	8 (5.67)	
1–3	0 (0.00)	13 (9.22)	
4–7	3 (33.33)	39 (27.66)	
8–11	2 (22.22)	63 (44.68)	
More than 11	2 (22.22)	17 (12.06)	
Not informed	0 (0.00)	1 (0.71)	

*Occupation*			0.17
Housewife	5 (55.56)	65 (46.10)	
Farmer	1 (11.11)	47 (33.33)	
Merchant	1 (11.11)	21 (14.89)	
Retired	1 (11.11)	6 (4.26)	
Student	1 (11.11)	2 (1.42)	

*Marital status*			0.27
Single	4 (44.44)	51 (36.67)	
Married	2 (22.22)	47 (33.33)	
Stable union	2 (22.22)	31 (21.99)	
Widow	0 (0.00)	10 (7.09)	
Separated	1 (11.11)	2 (1.42)	

*Ethnicity*			0.99
Asian race	0 (0.00)	1 (0.71)	
Caucasian	1 (11.11)	19 (13.48)	
Black	2 (22.22)	29 (20.57)	
Pardo	6 (66.67)	91 (64.54)	
Indigenous	0 (0.00)	1 (0.71)	

*Income*			0.60
1/2 salary	3 (33.33)	20 (14.18)	
1 salary	4 (4.44)	68 (48.23)	
2 salaries	1 (11.11)	31 (21.99)	
3 salaries	0 (0.00)	1 (0.71)	
More than 3 salaries	0 (0.00)	17 (12.06)	
Not stated	1 (11.11)	4 (2.84)	

*Smoking*			0.72
Yes	1 (11.11)	25 (17.73)	
No	7 (77.78)	108 (76.60)	
Former smoker	1 (11.11)	8 (5.67)	

*Alcoholism*			0.38
Yes	4 (44.44)	59 (41.84)	
No	4 (44.44)	78 (55.32)	
Former alcoholic	1 (11.11)	4 (2.84)	

**Table 2:** Lifestyle habits (açaí consumption, smoking, and alcoholism) among women who consumed açaí at least once a week.

Variables	*N*	%
*Years of consumption*		
Up to 10 years	7	4.66
>10 years	109	72.67
Not stated	25	16.67
No	9	6.00

*Form of consumption* ^*∗*^		
Juice	122	81.33
Pulp	19	12.67
Ice cream	6	4.00
No	9	6.00

*Weekly frequency*		
Once	42	28.00
Twice	19	12.67
Thrice	13	8.67
More than three times	60	40.00
Not stated	7	4.66
No	9	6.00

^*∗*^In this category, the participants were allowed to provide more than one answer.

**Table 3:** Relationship between the reproductive characteristics and açaí consumption among women residing in the Maracanã neighborhood, São Luís, MA.

Variables	Açaí consumption		*p*
	No *f* (%)	Yes *f* (%)	
*Age at menarche* ^*∗*^			0.88
10 years	0 (0.00)	5 (3.55)	
11–14 years	8 (88.89)	97 (68.80)	
More than 14 years	1 (11.11)	36 (25.53)	
Not stated	0 (0.00)	2 (1.42)	
No	0 (0.00)	1 (0.71)	

*Age at menopause* ^*∗*^			0.60
30 years	0 (0.00)	1 (0.71)	
35 years	0 (0.00)	3 (2.13)	
36 to 45 years	0 (0.00)	10 (7.09)	
Older than 45 years	3 (33.33)	19 (13.48)	
No	6 (66.67)	108 (76.60)	

*Full-term pregnancies* ^*∗*^			0.63
1	1 (11.11)	20 (14.18)	
2–4	3 (33.33)	75 (53.19)	
More than 4	2 (22.22)	27 (19.15)	
No	3 (33.33)	19 (13.48)	

*Preterm pregnancies* ^*∗∗*^			0.60
1	0 (0.00)	13 (9.22)	
No	9 (100.00)	128 (90.78)	

*Number of abortions* ^*∗*^			0.99
1	1 (11.11)	20 (14.18)	
2	0 (0.00)	2 (1.42)	
3	0 (0.00)	1 (0.71)	
4	0 (0.00)	1 (0.71)	
No	8 (88.89)	117 (82.98)	

*Breastfeeding* ^*∗∗*^			0.19
No	3 (33.33)	23 (16.31)	
Yes	6 (66.67)	118 (83.69)	

^*∗*^Chi-square test. ^*∗∗*^Fisher's exact test.

**Table 4:** Relationship between the results of mammography and breast ultrasound and açaí consumption among women residing in the Maracanã neighborhood, São Luís, MA.

Variables	Açaí consumption		*p*
	No *f* (%)	Yes *f* (%)	
*Mammography* ^*∗*^			0.20
Category I	2 (22.22)	34 (24.11)	
Category II	0 (0.00)	2 (1.42)	
Category III	0 (0.00)	3 (2.13)	
No	6 (66.67)	101 (71.63)	
Not stated	1 (11.11)	1 (0.71)	

*Ultrasound* ^*∗*^			0.96
Category I	1 (11.11)	35 (24.82)	
Categories I and II	0 (0.00)	1 (0.71)	
Category II	0 (0.00)	1 (0.71)	
Category III	0 (0.00)	2 (1.72)	
No	8 (88.89)	101 (71.63)	
Not stated	0 (0.00)	1 (0.71)	

**Table 5:** Relationship between comorbidities, previous history of cancer, presence of breast nodules, family history of breast cancer and other types of cancer, and açaí consumption among women residing in the Maracanã neighborhood, São Luís, MA.

Variables	Açaí consumption		*p*
	No *f* (%)	Yes *f* (%)	
*Family history of breast cancer* ^*∗∗*^			0.62
No	9 (100.00)	130 (92.20)	
Yes	0 (0.00)	11 (7.80)	

*Other types of cancer in the family* ^*∗∗*^			0.43
No	6 (66.67)	110 (78.01)	
Yes	3 (33.33)	31 (21.99)	

*Previous history of cancer* ^*∗∗*^			0.71
No	9 (100.00)	139 (98.58)	
Yes	0 (0.00)	2 (1.42)	

*Nodule* ^*∗∗*^			0.38
No	9 (100.00)	130 (92.20)	
Yes	0 (0.00)	11 (7.80)	

*Diabetes* ^*∗*^			0.91
Yes	1 (11.11)	19 (13.47)	
No	8 (88.89)	120 (85.11)	
Not stated	0 (0.00)	2 (1.42)	

*Heart disease* ^*∗∗*^			0.52
Yes	0 (0.00)	10 (7.09)	
No	9 (100.00)	131 (92.91)	

*Hypertension* ^*∗*^			0.96
Yes	3 (33.33)	31 (21.98)	
No	6 (66.67)	105 (74.47)	
Not stated	0 (0.00)	5 (3.55)	

^*∗*^Chi-square test. ^*∗∗*^Fisher's exact test.

## Data Availability

All data are included in the manuscript.
